# Targeting of GFP-Cre to the Mouse *Cyp11a1* Locus Both Drives Cre Recombinase Expression in Steroidogenic Cells and Permits Generation of *Cyp11a1* Knock Out Mice

**DOI:** 10.1371/journal.pone.0084541

**Published:** 2014-01-03

**Authors:** Laura O'Hara, Jean Philippe York, Pumin Zhang, Lee B. Smith

**Affiliations:** 1 Medical Research Council Centre for Reproductive Health, University of Edinburgh, Edinburgh, United Kingdom; 2 Department of Molecular Physiology and Biophysics, Baylor College of Medicine, Houston, Texas, United States of America; Louisiana State University Health Sciences Center, United States of America

## Abstract

To permit conditional gene targeting of floxed alleles in steroidogenic cell-types we have generated a transgenic mouse line that expresses Cre Recombinase under the regulation of the endogenous Cytochrome P450 side chain cleavage enzyme (*Cyp11a1*) promoter. Mice Carrying the *Cyp11a1-GC* (GFP-Cre) allele express Cre Recombinase in fetal adrenal and testis, and adrenal cortex, testicular Leydig cells (and a small proportion of Sertoli cells), theca cells of the ovary, and the hindbrain in postnatal life. Circulating testosterone concentration is unchanged in *Cyp11^+/GC^* males, suggesting steroidogenesis is unaffected by loss of one allele of *Cyp11a1*, mice are grossly normal, and Cre Recombinase functions to recombine floxed alleles of both a YFP reporter gene and the Androgen Receptor (AR) in steroidogenic cells of the testis, ovary, adrenal and hindbrain. Additionally, when bred to homozygosity (*Cyp11a1^GC/GC^*), knock-in of GFP-Cre to the endogenous *Cyp11a1* locus results in a novel mouse model lacking endogenous *Cyp11a1* (P450-SCC) function. This unique dual-purpose model has utility both for those wishing to conditionally target genes within steroidogenic cell types and for studies requiring mice lacking endogenous steroid hormone production.

## Introduction

Mouse models of conditional gene ablation using the Cre/loxP system [1] have led to a deeper understanding of cell-specific gene function, as well as novel models of development and disease. The power of this system relies upon temporal or spatial localisation of Cre Recombinase expression, with different promoters able to drive Cre Recombinase expression in restricted cell lineages or time-points. Over 500 Cre expressing mouse lines are now available [2], making the choice of which Cre line to use target a particular cell-type ever more important. As such, a measure of the utility of any Cre Recombinase line is dependent upon validation of its sites and timing of expression [3].

Steroidogenic enzymes are produced by the Leydig cells of the testis in the male, the theca cells of the ovary in the female, the trophoblastic giant cells of the placenta and the adrenocortical cells of the adrenal gland in both sexes [4]. Steroidogenic enzyme expression has also been detected in the brain in several species [5], [6]. Cytochrome P450 side chain cleavage (P450-SCC), the product of *Cyp11a1* gene expression, plays a major role in the control of steroidogenesis by mediating the conversion of cholesterol to pregnenolone. As the first step in the synthesis of all steroidogenic enzymes it is therefore common to all steroidogenic cells. Consequently transgenic disruption of the *Cyp11a1* locus results in mice that fail to synthesise steroids, and die shortly after birth due to adrenal insufficiency, unless rescued with exogenous steroids [7]. *Cyp11a1* knockout mice display a variety of aberrant phenotypes associated with various steroid hormone deficiency syndromes and as such provide an excellent model for investigation of steroid function [8].

Additionally, the requirement for *Cyp11a1* expression in all steroidogenic cells makes this locus an ideal candidate to drive Cre recombinase expression to target recombination of floxed genes specifically within these cells. Indeed two Cyp11a1-Cre lines have previously been generated, one using a 4.4 Kb promoter fragment of human *CYP11A1* to drive Cre expression in the steroidogenic cells and brain [9], and one using a BAC transgenic approach to place Cre Recombinase under the control of an extended sequence of the mouse *Cyp11a1* promoter [10]. As our primary interest is in the process of steroidogenesis we have taken an alternative approach, targeting a GFP-Cre transgene into the endogenous mouse *Cyp11a1* locus. In contrast to the published Cyp11a1-Cre mouse lines, this approach permits generation of *Cyp11a1* knockout mice, in addition to driving Cre Recombinase expression in steroidogenic cells of the testis, ovary, adrenal and hindbrain.

In this paper we describe the functional characterisation of this novel model, highlighting its utility both for targeting Cre Recombinase to steroidogenic cell-types and for generation of mice lacking *Cyp11a1*.

## Materials and Methods

### Generation of Cyp11a1-GC Transgenic Mice

A 7.9 kb genomic fragment of *Cyp11a1* (containing the first 4 exons) was gap repaired by homologous recombination onto pDTA.4B from a BAC clone obtained from Sanger Institute. A targeting vector containing GFP-Cre cassette (GC) and FRT site flanked Kanamycin/neomycin selection cassette was used to disrupt the exon containing the ATG translation start-site. The construct was electroporated into 129P2/OlaHsd derived E14Tg2a.4 embryonic stem (ES) cells [11]. Correctly targeted ES cells were injected into recipient blastocysts. The resulting chimeric animals were crossed to C57BL/6 mice. Genomic DNA isolated from wild-type and heterozygous mice were digested with EcoRV, separated in 0.8% agarose gel, transferred to charged nylon membrane, and hybridized with ^32^P-labeled probes designed to be located either upstream (5′) or downstream (3′) of the transgene insertion site. Due to EcoRV sites present in the transgene, a band of 14 kb could be seen in wild-type mice with both 5′ and 3′ probes. Heterozygotes showed a band of 14 kb and also 10.5 kb for the 5′ or 6.5 kb for the 3′ probe.

### Production of Cyp11a1-KO, Cyp11a1-GC ARKO and Cyp11a1-GC EFYP reporter mice

To generate *Cyp11a1* knockout mice, *Cyp11a1^+/GC^* male and female were inter-crossed. For lineage tracing of Cre Recombinase function, *Cyp11a1^+/GC^* male mice were bred to R26R-EFYP homozygous females [12]. To ablate androgen receptor from steroidogenic cells, male *Cyp11a1^+/GC^* mice were mated to C57BL/6J female homozygous *AR^fl/fl^* mice [13]. The *Cyp11a1^+/GC^*:AR*^fl/y^* male offspring from these matings were termed ‘Cyp11a1-ARKO’, whereas the *Cyp11a1^+/+^*:AR*^fl/y^* littermates were used as controls, termed ‘control’. Sex and genotype ratios were identified at the expected Mendelian ratios across all matings. All mice were bred under standard conditions of care and use under licensed approval from the UK Home Office (PPL: 60/4200).

### Recovery of tissues

Adult mice were culled at post-natal day (d) 100 by inhalation of carbon dioxide and subsequent cervical dislocation. Body weight and anogenital distance were measured and mice were examined for any gross abnormalities of the reproductive system. Testes and SV were removed and weighed from male mice. Ovaries were removed from females. Tissues were fixed in Bouin's fixative (Clin-Tech, Guildford, UK) for 6 h. Bouin's-fixed tissues were processed and embedded in paraffin wax, and 5-µm sections were used for histological analysis as reported previously [14]. Sections of testis were stained with hematoxylin and eosin using standard protocols and examined for histological abnormalities.

### PCR genotyping

PCR genotyping for inheritance of the Cyp11a1-GC allele was carried out as previously described http://jaxmice.jax.org/protocolsdb/f?p=116:2:3453436993967916::NO:2:P2_MASTER_PROTOCOL_ID,P2_JRS_CODE:3815,010988 using primers P450-F: 5′ GAGCTGCCTGCCAGTGTTTG 3′; P450-R: 5′ GGACCTAGGACTGCTAGTAG 3′; and GFP-R: 5′ GTCCAGCTCGACCAGGATGG 3′. PCR amplification products were resolved using a QiaXcel capillary system (Qiagen, UK).

### Determination of genomic ablation of AR

Frozen tissues were genotyped for genomic ablation of AR as previously described [15]. PCR amplification products were resolved using a QiaXcel capillary system (Qiagen, UK). An amplicon of 1142 bp indicated presence of a floxed AR whilst an amplicon of 612 bp indicated recombination between *loxP* sites and deletion of AR exon 2.

### RNA extraction and reverse transcription

RNA was isolated from frozen adrenal glands collected from e16.5 to d0 offspring of Cyp11a1^+/GC^ x Cyp11a1^+/GC^ matings using the RNeasy Mini extraction kit with RNase-free DNase on-column digestion (Qiagen, Crawley, UK) according to the manufacturer's instructions. RNA was quantified using a NanoDrop 1000 spectrophotometer (Thermo Fisher Scientific, Waltham, MA, USA). cDNA was prepared using the SuperScript® VILO™ cDNA Synthesis Kit (Life Technologies) according to manufacturer's instructions.

### RT-PCR validation of Cre Recombinase gene expression

RT-PCR was performed for *Cre* on adrenal cDNA using primers – forward: GATCGCTGCCAGGATATACG, reverse: AGGCCAGGTATCTCTGACCA, synthesised by Eurofins MWG Operon. PCR amplification products were resolved using a QiaXcel capillary system (Qiagen, UK). An amplicon of 400 bp indicated presence of Cre transcript.

### Quantitative analysis of *Cyp11a1* gene expression

Quantitative RT-PCR was performed for *Cyp11a1* on adrenal cDNA using primers – forward: AAGTATGGCCCCATTTACAGG, reverse: TGGGGTCCACGATGTAAACT, synthesised by Eurofins MWG Operon and probe 104 from the Roche Mouse Universal Probe library (Roche, Welwyn, UK). Assays were carried out using the ABI Prism 7900 Sequence Detection System (Applied Biosystems). The expression of each gene was related to an internal housekeeping gene assay for *Actb* (Roche, Welwyn, UK).

### Fluorescence microscopy

Freshly dissected organs were visualised with a Leica MZFLIII microscope and an epifluorescent YFP filter. Photographs were taken with a Photometrics CoolSNAP camera and PMCapture Pro 6.0 software.

### Histochemical Analysis

Double immunofluorescent staining for YFP and 3βHSD was performed on Cyp11a1-GC:EYFP and littermate controls and single immunohistochemical staining for AR was performed on Cyp11a1-Cre ARKO mice and littermate controls.

YFP/3βHSD immunofluorescence was performed based on a double immunofluorescence protocol described previously [15]. Sections were deparaffinized and rehydrated, and high-pressure antigen retrieval was performed in 0.01M pH 6 citrate buffer for 5 minutes. Endogenous peroxidase was blocked using 3% hydrogen peroxide in methanol and non-specific antibody binding sites were blocked with normal chicken serum (NChS)/TBS/BSA. Sections were incubated overnight at 4°C with rabbit anti-GFP/YFP (Abcam ab6556) then YFP immunostaining was detected using chicken anti-rabbit peroxidase (Santa Cruz sc-2963) diluted in NChS/TBS/BSA incubated for one hour at room temperature, then fluorescein Tyramide Signal Amplification system (‘TSA™’, Perkin Elmer) to manufacturer's instructions for 10 minutes at room temperature. Antigen retrieval was then repeated and the same slides stained using goat anti-3βHSD antibody (Santa Cruz sc-30820) overnight at 4°C and detected with chicken anti-goat peroxidase (Santa Cruz sc-2953) diluted in NChS/TBS/BSA incubated for one hour at room temperature and TSA of a different colour to distinguish from the first antibody detection. Slides were counterstained with Sytox Green (Sigma, UK, #P4170), mounted with PermaFluor mounting medium (Thermo Scientific, UK). Images were captured using a LSM 710 confocal microscope (Zeiss) with Zen software.

Automated AR immunostaining was performed on a Bond-max machine (Leica, UK) using rabbit anti-AR (Abcam ab74272) at 1∶250 concentration and a Polymer Refine Detection kit according to the manufacturer's instructions. Antigen retrieval was performed with Leica Bond ER2 buffer. Slides were counterstained with hematoxylin, dehydrated, and mounted with Pertex (Histolab, Gothenburg, Sweden); images were captured using a Provis AX70 microscope (Olympus, Southend-on-Sea, UK) equipped a Axiocam HRc (Zeiss, Welwyn Garden City, UK).

A minimum of five mice of each genotype was used and sections from transgenic and control littermates were processed in parallel on the same slide, on at least two separate occasions. A no-primary control was included to ensure that any staining observed was specific.

### Quantification of AR ablation in Leydig cells

AR in Leydig cell was quantified using a method based on a previously published protocol [16]. Briefly AR ablation was quantified in testis sections immuno-stained for 3βHSD and AR from three Cyp11a1-ARKO and two control animals at d100. Sections were analysed using Image-Pro Plus 6.2 software with a Stereology 5.0 plug-in (Media Cybernetics U.K., Berkshire, UK) with the ×63 objective on a Leitz DBRB microscope fitted with a Prior Pro-Scan automatic stage (Prior Scientific Instruments Ltd., Cambridge, UK). The Count (NV) setting was used to count all 3βHSD-positive cells staining either positive or negative for AR.

## Results

### Generation of *Cyp11a1-GC* mice

**Figure 1:**
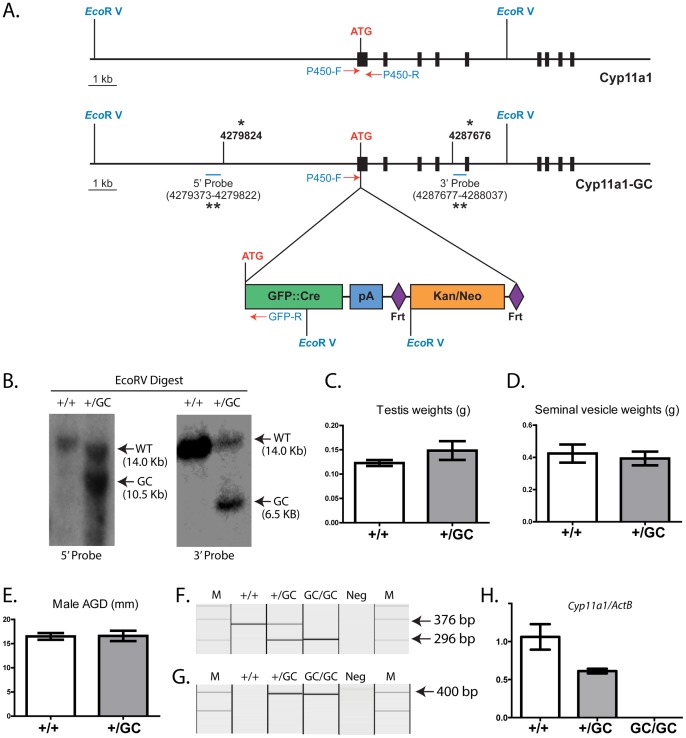
Generation of the *Cyp11a1-GC* line and production of mice lacking *Cyp11a1*. A 7.9*Cyp11a1* (containing the first 4 exons) was gap repaired onto pDTA.4B from a BAC clone obtained from Sanger Institute. GFP::Cre plus the neomycin resistant gene (flanked by Frt sites; also confers kanamycin resistance in bacteria) was inserted into the first ATG site via homologous recombination. Electroporation of ES cells was followed by neomycin selection and genotyping to confirm recombination of the Cyp11a1-GC construct into the endogenous Cyp11a1 locus (1a) Note, EcoRV recognition sites, Southern blot probe binding sites and PCR genotyping primer binding sites are shown. Asterisks identify base-pairs from chromosome 9 in build 37.1 of the mouse ensembl database (www.ensembl.org). Correctly targeted clones were identified by Southern blotting using 5′ and 3′ probes following digest of Genomic DNA with EcoRV (1b). Average testis weight (1c), seminal vesicle weight (1d) and AGD (1e) do not differ between *Cyp11a1^+/+^* and *Cyp11a1^+/GC^* mice. PCR genotyping of offspring from inter-crossed *Cyp11a1^+/GC^* mice identified inheritance of the *Cyp11a1-GC* allele (296 bp; 376 bp = WT) (1f) RT-PCR analysis on adrenals taken from these pups demonstrated expression of Cre Recombinase (400 bp) (1 g), and absence of endogenous *Cyp11a1* transcript (1 h), as predicted. Together these data confirmed the utility of the line for production of mice lacking *Cyp11a1*. M = marker; Neg =  no template control.

A 7.9 Kb genomic fragment of *Cyp11a1* (containing the first 4 exons) was gap repaired onto pDTA.4B from a BAC clone obtained from the Sanger Institute (Cambridge, UK). GFP::Cre plus a neomycin resistant gene (also confers kanamycin resistance in bacteria) flanked by Frt sites was inserted into the first ATG site via homologous recombination as previously described [11]. The targeted allele was named *Cyp11a1-GC* (Figure 1a). The construct was electroporated into 129P2/OlaHsd derived E14Tg2a.4 embryonic stem (ES) cells [11]. Correctly targeted ES cells were identified through Southern blotting following EcoRV digest of ES cell DNA (Figure 1b), and were injected into recipient blastocysts. The resulting chimeric animals were crossed to C57BL/6 mice to generate a transgenic line containing the *Cyp11a1-GC* allele (*Cyp11a1^+/GC^*).

### Production of *Cyp11a1* Knock Out mice

*Cyp11a1^+/GC^* mice were normal in appearance and healthy. No significant difference in anogenital distance (AGD), testis weight or seminal vesicle (SV) weight between *Cyp11a1^+/GC^* and *Cyp11a1^+/+^* males was noted (Figure 1c to e), demonstrating androgen production was unaffected. Male and female *Cyp11a1^+/GC^* mice were inter-crossed to generate *Cyp11a1^GC/GC^* animals, and genotyped confirmed by PCR (Figure 1f). RT-PCR analysis of adrenal tissue demonstrated production of Cre recombinase by both *Cyp11a1^+/GC^* and *Cyp11a1^GC/GC^* mice (Figure 1g) and quantitative RT-PCR for *Cyp11a1* confirmed that *Cyp11a1^GC/GC^* mice lack endogenous *Cyp11a1* (Figure 1h), confirming the utility of this line for generation of *Cyp11a1*-null mice.

### Expression of GFP and Cre Recombinase

**Figure 2:**
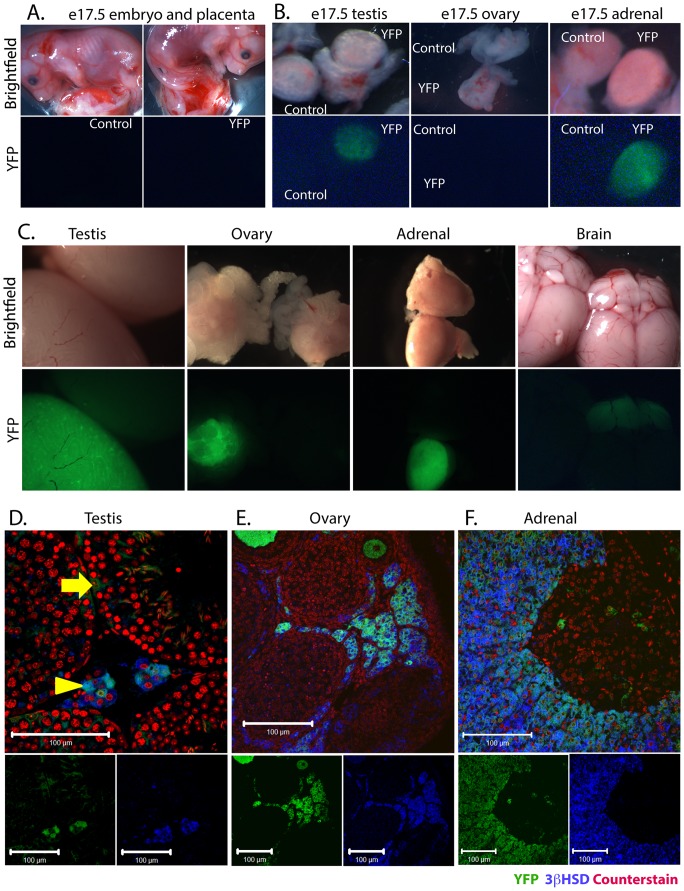
Expression of YFP in tissues from *Cyp11a1^+/GC^*:R26-EYFP mice. Bright field and epifluorescence localisation of YFP expression in e17.5 embryos and placenta. YFP expression is undetectable in the placenta (2a). Consistent with predicted expression pattern of *Cyp11a1*, YFP expression is detectable in the fetal testis and adrenal, but absent from the fetal ovary (2b). Epifluorescence microscopy in adulthood confirms expression of YFP in the testis, ovary, adrenal of both sexes and cerebellum (2c). Immunofluorescent localisation of YFP using an anti-YFP antibody on tissues sections demonstrates YFP expression localised to testicular Leydig cells (arrowhead), and a small number of Sertoli cells (arrow) (2d) Bar = 100 µm. In the ovary, YFP expression is restricted to theca cells (2e) Bar = 100 µm. In the adrenal YFP is localised to the adrenal cortex (2f) Bar = 100 µm.

Although the *Cyp11a1-GC* allele contains GFP, no GFP expression could be detected in tissues when visualised under fluorescence microscopy, or via immunohistochemistry, at any age examined (data not shown). To establish the utility of the *Cyp11a1-GC* allele as a Cre Recombinase expressing line *Cyp11a1^+/GC^* males were bred to homozygous R26R-EYFP females [12]. Cre-mediated recombination removes an upstream stop codon, permitting persistent expression of YFP in cells which acts as a lineage tracer for sites of functional Cre Recombinase expression. Examination of e17.5 embryos under fluorescence microscopy revealed YFP expression restricted to the fetal testis and adrenal (of both sexes) (Figure 2 a, b). Consistent with established sites of expression of *Cyp11a1* in the embryo, YFP expression was not detected in the fetal ovary or placenta. In a divergence from the reported expression pattern of Cre Recombinase under the control of 4.4 Kb of the human *Cyp11a1* promoter, YFP expression was absent from the fetal brain when examined by fluorescence microscopy. Furthermore, as predicted, no expression was observed in other tissues of the body, including pituitary, kidney, heart, liver, seminal vesicles and pancreas (figure 2a and data not shown).

YFP expression was also absent from the ovary when examined at postnatal day 12 (d12) whilst YFP expression continued to be observed in the testis and adrenal (data not shown). In adulthood, YFP expression was again restricted to steroidogenic tissues, not only the testis and adrenal (both sexes), but also in the ovary and cerebellum (Figure 2c). Double immunofluorescent detection of YFP on tissue sections combined with 3βHSD to mark steroidogenic cells localised YFP expression to a subset of Leydig cells of the testis (and occasional Sertoli cells), theca cells of the ovary and the cortex of the adrenal, consistent with the known expression pattern of *Cyp11a1* (P450-SCC) (Figure 2 d–f).

### Validation using a second floxed line

**Figure 3:**
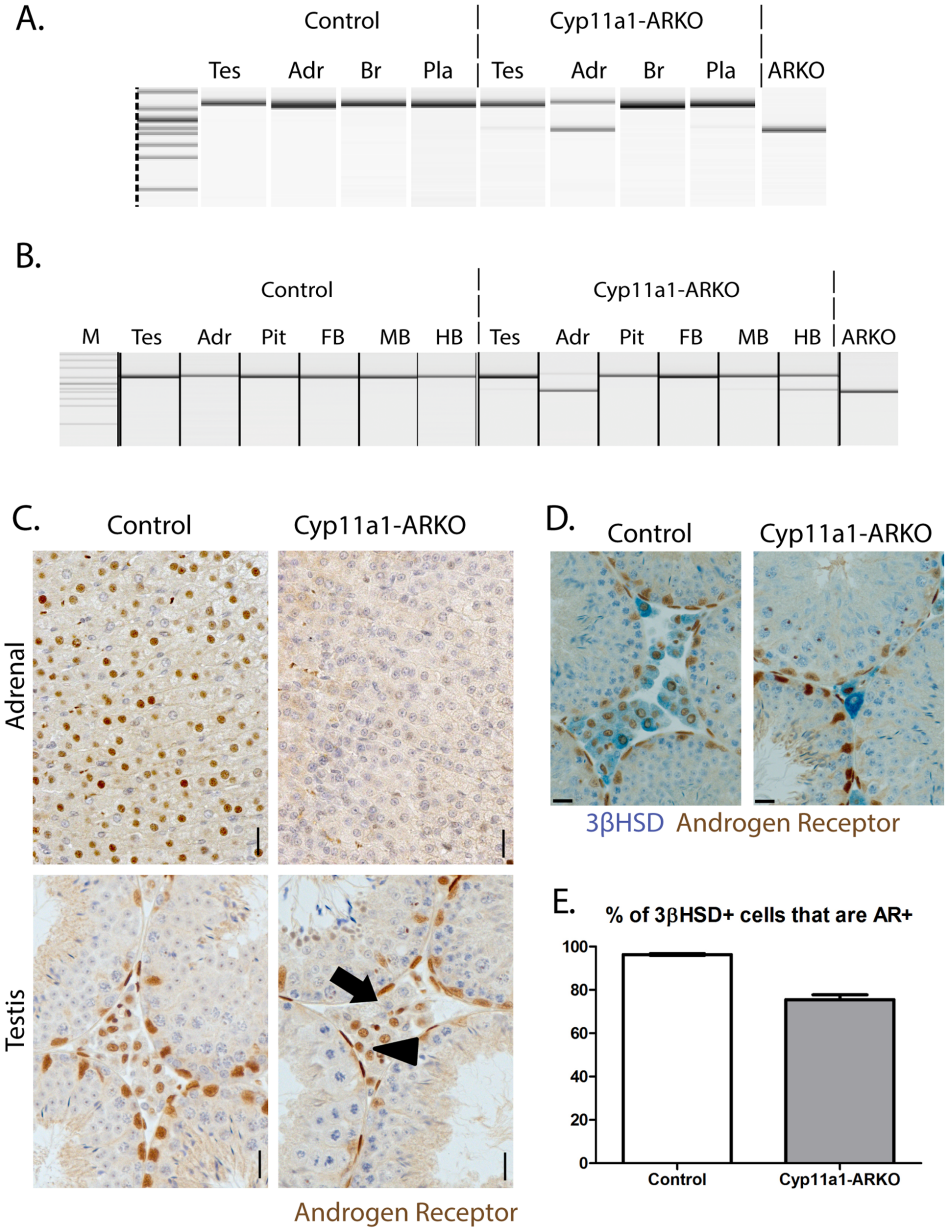
Characterisation of *Cyp11a1^+/GC^*:ARKO mice. PCR interrogation of genomic DNA from tissues of male e17.5 embryos demonstrates recombination of the floxed AR allele in testis and adrenal, but not brain or placenta. Amplification from total ARKO mouse included for comparison (3a). PCR amplification on adult tissues confirms ablation of AR form a proportion of testis and adrenal cells, and in addition, confirms ablation of AR from hindbrain (HB), but not forebrain (FB) or midbrain (MB) (3b). Immunohistochemical localisation of AR on adult adrenal and testis sections confirms absence of AR expression from the adrenal cortex of Cyp11a1-ARKO mice, and from a proportion of testicular Leydig cells (arrow) although a proportion of Leydig cells retain AR expression (arrowhead) (3c) Bar = 20 µm. Double staining for 3βHSD and AR identifies interstitial Leydig cells (3d) allowing quantification of AR ablation in Leydig cells of the Cyp11a1-ARKO (3e).

To determine the functional ability of the *Cyp11a1-GC* allele to excise an independent floxed gene, located at a distinct chromosomal locus *in vivo*, *Cyp11a1^+/GC^* male mice were bred with females carrying a floxed allele of the X-linked Androgen Receptor (*AR^fl/fl^*) [13]. Male pups were examined. Recombination of AR genomic DNA was interrogated at e17.5. Consistent with observations of recombination at the R26R-EFYP locus, recombination was detected in a proportion of testis cells and adrenal cells, but completely absent from the placenta and brain (Figure 3a). In adulthood, recombination was detected in a small proportion of cells of the testis and the majority of the adrenal, along with recombination in the hindbrain (but not the forebrain or midbrain) (Figure 3b). Immunohistochemical localisation of AR in tissues sections from adult adrenals and testes showed recombination of AR throughout the adrenal cortex, and in a proportion of testicular Leydig cells (Figure 3c), again consistent with the data derived from recombination of the R26R-EYFP locus, and the normal cellular localisation of *Cyp11a1* (P450-SCC). AR/3βHSD double staining (Figure 3d) and stereological cell counting quantified that 96% of 3βHSD Leydig cells in littermate control testes express AR compared to 76% in Cyp11a1-ARKO testes (Figure 3e).

## Discussion

We describe the construction and functional validation of a novel dual-purpose mouse line of utility for the study of steroidogenesis and steroidogenic cell types. Targeting of Cre Recombinase into the mouse endogenous *Cyp11a1* locus, permits both the generation of *Cyp11a1* knockout mice and a novel Cre Recombinase line targeting steroidogenic cell-types. This line has been deposited with the Jackson Laboratory, (Maine, USA) stock number 010988.

Gene expression and functional recombination by Cre Recombinase is restricted to the developing cortex of the fetal adrenal (both sexes) and Leydig cells of the fetal testis in prenatal life. Postnatally, in addition to the adrenal and Leydig cells (and a small proportion of Sertoli cells) in the testis, Cre Recombinase-mediated recombination is detectable in theca cells of the ovary, and the hindbrain. Taken together, the Cyp11a1-GC line provides a novel Cre Recombinase model with utility for targeting cells of steroidogenic lineages. Despite the presence of GFP in the transgene, no GFP expression was noted in Cyp11a1-GC tissues by either epifluorescence or immunofluorescence and a *Cyp11a1^+/GC^*:R26-EYFP line was required to localise Cre recombination. This may be because low levels of the transgene do not produce enough GFP to be detectable by immunofluorescence, but the same level of Cre is enough to result in recombination in a proportion of cells. This requires further investigation but nevertheless does not appear to detract from the function activity of Cre Recombinase and the utility of the line as such.

Several Cre Recombinase lines now target the steroidogenic lineage [9], [10], [17], each with overlapping but not identical expression patterns (Table 1), thus providing a level of choice and refinement when targeting floxed genes in steroidogenic cells. Two other published models utilise a *Cyp11a1* promoter as a driver of Cre Recombinase expression. The first exploits 4.4 Kb of the human *CYP11A1* promoter in a minigene construct introduced by pronuclear injection (SCC-Cre) [17]. Whilst the resultant expression of Cre Recombinase (as defined by lineage tracing) closely mimics that of the Cyp11a1-GC mouse, there are several distinct differences. Expression of SCC-Cre is detected albeit weakly in the fetal ovary (endogenous Cyp11a1 is not found in the fetal ovary), and in distinct regions of the brain in both fetal and adult life, conversely no fetal ovary expression is detected in the Cyp11a1-GC line, and expression in the brain is restricted to postnatal life, and in the hindbrain, a localisation distinct to that of expression in the SCC-Cre brain [17], [18].

**Table 1:** Overview of Cyp11a1-Cre Recombinase lines.

Mouse line	SCC-Cre [9]	Cyp11a1-iCre [10]	Cyp11a1-GC
Nature of transgene	4.4 Kb – human promoter (pronuclear)	BAC – mouse promoter (pronuclear)	Knock-in to mouse *Cyp11a1* locus
**Prenatal Placenta**	−	?	−
**Prenatal Adrenal**	+++	+++	+++
**Prenatal Testis**	+++	+++	+++
**Prenatal Ovary**	+	−	−
**Prenatal Brain**	++	?	−
**Postnatal Adrenal**	+++	+++	+++
**Postnatal Testis**	+++	+++	+++
**Postnatal Ovary**	+++	+++	+++
**Postnatal Brain**	++	?	++
**Cyp11a1-KO?**	No	No	Yes

(+ = expressed; − = not expressed; ? = expression not reported).

A second line, Cyp11a1iCre utilised a mouse BAC transgenic approach to drive Cre recombinase expression utilising an extensive region of the mouse promoter (again introduced by pronuclear injection) [10]. Lineage tracing of the Cre Recombinase expression closely mimics that of Cyp11a1-GC allele, suggesting that inclusion of a more extensive promoter region, and perhaps from mouse rather than human, provides a more faithful representation of endogenous gene expression. Nevertheless, all three lines have utility for targeting steroidogenic cell-types.

The unique advantage of the Cyp11a1-GC line over other steroidogenic Cre Recombinase lines is the ability to also generate *Cyp11a1* knock out mice. Ablation of *Cyp11a1* in the original *Cyp11a1* knockout mouse has provided important insights into steroid hormone production and action, providing insight into human conditions of steroid hormone deficiency [19]. The dual nature of the Cyp11a1-GC allele is the ability not only to ablate P450-SCC function, but also to exploit the knock-in of Cre Recombinase to simultaneously target other floxed genes in steroidogenic cells of the *Cyp11a1* knockout mouse, and/or to utilise lineage tracing to track the fate of these cells. Both of these approaches will enhance our understanding of the control of steroidogenesis.
